# A Trip through Europe and the Hospitals

**Published:** 1915-03

**Authors:** L. C. Fischer

**Affiliations:** Atlanta, Ga.


### Journal-Record of Medicine

Successor to Atlanta Medical and Surgical Journal, Established 1855
and Southern Medical Record, Established 1870

**OWNED BY THE ATLANTA MEDICAL JOURNAL COMPANY**

PUBLISHED MONTHLY

*Official Organ Fulton County Medical Society, State Examining Board, Presbyterian Hospital, Atlanta, Birmingham and Atlantic Railroad Surgeons' Association, Chattahoochee Valley Medical and Surgical Association, Etc.*

EDGAR BALLENGER, M. D., Editor
BERNARD WOLFF, M. D., Supervising Editor
A. W. STIRLING, M. D., C. M., D. P. H.; J. S. HURT, B. Ph., M.D.
GEO. M. NILES, M. D., W. J. LOVE, M. D., (Ala.); Associate Editors
R. R. DALY, M. D., Associate Editor
E. W. ALLEN, Business Manager

**COLLABORATORS**

W. F. WESTMORELAND, M. D., General Surgery
F. W. McRAE, M. D., Abdominal Surgery
H. F. HARRIS, M. D., Pathology and Bacteriology
E. B. BLOCK, M. D., Diseases of the Nervous System
MICHAEL HOKE, M. D., Orthopedic Surgery
CYRUS W. STRICKLER, M. D., Legal Medicine and Medical Legislation
E. C. DAVIS, A. B., M. D., Obstetrics
E. G. JONES, A. B., M. D., Gynecology
R. T. DORSEY, Jr., B. S., M. D., Medicine
L. M. GAINES, A. B., M. D., Internal Medicine
GEO. C. MIZELL, M. D., Diseases of the Stomach and Intestines
L. B. CLARKE, M. D., Pediatrics
EDGAR PAULLIN, M. D., Opsonic Medicine
THEODORE TOEPEL, M. D., Mechano Therapy
A. W. STIRLING, M. D., Etc., Diseases of the Eye, Ear, Nose and Throat
BERNARD WOLFF, M. D., Diseases of the Skin
E. G. BALLENGER, M. D., Diseases of the Genito-Urinary Organs

VOL. LXI ATLANTA, GA., MARCH, 1915. NO. 12

### A TRIP THROUGH EUROPE AND THE HOSPITALS.

BY DR. L. C. FISCHER, ATLANTA, GA.

(Continued.)

After having watched the clinics at Berne for several days and studied goiter until we waked at night almost choking, having dreamed that we were afflicted with bulging eyes and a mass that would extend down level with the clavicles, we left there for Vienna on July 7th. It sounds strange to say that it was a cold, disagreeable day, so much so that an overcoat was imperative. We were soon out in the Alps on our way to Innsbrook, the capital of Tyrol. There were many things that I had heard of and associated with the Alps all of my life, among them was the Alpine Rose, the Eidelweise and the wonderful Swiss cattle. Was very much disappointed to see that the Alpine rose is not a rose at all, but is a flower somewhat similar to our mountain laurel and grows on low stubby bushes. The flower is a beautiful pink without odor. As soon as it is picked it wilts. We were not permitted to pull an Eidelweise as they grow from seven to nine thousand feet up on the sides of the mauntains above the timberline. It is a queer looking little posie, star-shaped. The petals are covered by a soft, velvety fiber resembling cotton. We saw some of the wonderful watch dogs that roam the mountains and occasionally find some mountain climber who has lost his foothold or slipped on a glacier. With these stories told us constantly we had no inclination to climb the dizzy heights of the Alps. We saw the little chamois goats about which so many stories are told of their winding their horns around the limbs of trees and swinging across a precipice from one mountain to another. (This is one of the many stories that is told to tourists with an absolutely straight face. They have found out that there are so many quite wonderful things that are really true that they are at liberty to tell most any story that happens to come to their mind at the time, such as the wonderful story of William Tell, etc). They are much smaller than our goats and are rapidly becoming extinct, except in the large hunting preserves of the government. The Swiss cattle are really wonderful. We saw them in droves of hundreds feeding on the sides of the mountains, and as a rule near the timberline, for during the summer months they are all kept in the mountains, the milk being made into cheese and butter and sent down to customers in the settlements. No cattle are kept in the valleys during the summer months, for at this time the men, women and children are making hay and storing it for the bitter cold that they have coming on as early as September and lasting often until May. All along the sides of the mountains and in the valleys are the little hay barns and homes of the farmers. A great many of the barns and houses are covered with rock, this partly necessary for the lack of timbers, then too, the people there build their homes to last for centuries. We tried for hours to locate the cattle barns; finally discovered that they lived in the houses with the people; that in one story buildings the cows lived in one end and the people in the other; and that in two story structures the cows lived on the ground floor. It was explained to us that they did this for the reason that the animal heat from the cattle helps to warm their rooms and save fuel. There is practically no farming in the whole of Switzerland except raising feed for the cattle, vegetables and fruits. Butter, milk and cheese are their principal products. The manufacture of jewelry is by no means a lost art. They are said to make the finest watches in the world. Then too mining is quite an industry.

During our travels through Switzerland it was invariably an effort to make ourselves understood in any language. They have no distinct tongue of their own in that they are bounded on every side by people speaking Italian, French and German, and close to the borders the people speak in broken manner the language of the people nearest them, and in the center, as Berne, they have a dialect entirely their own. After stumbling over all the foreign words in your vocabulary in an effort to give instructions as to the train desired, or destination, or the best hotel in the next city, having thought you were doing fine speaking these various languages, it was often surprising to hear the porter reply and speak in better English than you would use. On one occasion we thought we were late for the train, but were positive that we were getting the wrong one. After making use of all the various sign languages at our command, the porter said quietly, "Yes, you are taking the train for Zurich." It was told me on an American doctor who was visiting Berne during the Clinical Congress, that he tried in four different languages to ask for a cigar. When finally in desperation he said to the young lady clerk, "Give me a cigar," the young lady's reply was, "Why in thunder didn't you say that first?"

We were in Berne on the "Fourth of July." All the Americans there were fortunate to procure small flags of the stars and stripes. You would meet them in every block and each one seemed to be a little prouder and show his independence a little more than the day before. This was also a national holiday in Switzerland. Imagine our delight when the bands were playing "My Country, 'tis of Thee." While this is as much their air as ours, it seemed to belong more to us in this foreign land.

From the time that the Crown Prince was killed, while we were in Italy, there were constant and persistent rumors of war, and as we were getting nearer Austria, rumors increased. In Switzerland they were preparing their army and drilling recruits. Switzerland has a very small standing army and not enough to resist any power. Her soldiers are mostly hired to other nations. On the Fourth of July we saw great armies of them drilling and taking trains for their stations. They reminded one very much of a crowd of country school boys who had never before handled a gun. They came from all stations of life. The soldiers of the rank and file are as a rule from the laboring class; the officers, from the higher strata and educated people. While the Swiss soldiers are untrained men and not supposed to fight except where employed by other nations, they have proven themselves to be some of the bravest men in the world. The Swiss Guard at Rome who stand watch over the Pope are the type of their soldiers. It is said that the reason the Roman Catholic Church employs the Swiss Guard at the Vatican is that they can not be bribed, and the Pope is perfectly safe in their care.

Traveling for hours, we came to Zurich, one of the greatest university towns of Europe, and from there went on to Innsbrook, where we expected to spend some time; but on account of the cold and snow spent only one night. By reason of our inability to speak the language of these people, we paid cab fare from the depot to our hotel, one block. We were surprised to find upon going to our hotel, that instead of sleeping on a feather bed it was placed on top and used as a comfort. It is true in the whole of Europe, expressed in the present day parlance, that they "see you coming," and they believe in living by the golden rule of David Harum, "Do the other fellow and do him first."

We left Innsbrook the following morning for Vienna, Austria, which is a long, tiresome ride. Even though it was the eighth of July, we were most uncomfortable with cold. Several times our train went as high as 3,500 feet up the mountain, often with two and occasionally three engines pulling us. For an hour we were in a heavy snow storm. While trees and shrubbery in the mountains were green, they were covered with snow. It was explained to us that even though the snow did stay for some time, it did not injure the vegetation. On our way we crossed the historical river Po and then approached what is often described as the beautiful "blue Danube." It was at the rainy season and both of these which are said to be such beautiful streams, were now nothing but mad rushing torrents of muddy water. They are both formed by the snow of Switzerland and surrounding country, the Danube especially being very swift and, while navigable, the boats go best down stream. Going up the speed is very slow, and, at this season of the year, almost impossible. The Danube flows through Vienna, Budapest and Belgrade. We expected to take this trip, but before we were able to do it, the Austrians were bombarding the Servians, and we decided that a pleasure craft would be one that would have the bow turned towards America and Atlanta. Never was a fond memory of the "Red hills of old Georgia," so dear as at certain times on this trip. It seemed to me that even razor back hogs and scrubby cattle far exceeded in beauty any of the wonderful specimens we had seen in Europe.

The farming and general conditions throughout Austria varied very little from what we had seen in Italy and Switzerland. The individual farms are small, never over a few acres; but the land is cultivated very scientifically and the yields are from three to five times that of our farms. This is especially true with grain. They raise a great deal of wheat and rye, and especially the latter. It was interesting to notice that their farm implements are about the same as the ones used hundreds of years ago; wooden pitch forks and plow stocks of the same material. The grain in many instances was cut with a scythe blade, the reaper being often on his knees and cutting inwards allowing the grain to fall on that that was standing. Following him were several women and children taking this up and making it into small bundles. The women, children and old men do the greatest amount of work, while the young and healthy men are in the army and the general employment of the government. As the government owns all of the railroads, street cars, etc., there are naturally a great many men who fill these various positions. Throughout the farming districts it was seldom that we saw a horse, and never a mule. They, the same as Italians, plow a great many oxen, milk cows and donkeys.

We finally reached Vienna on the night of the eighth of July, which was dark and rainy. Had quite a time convincing the customs officer, in a language that was entirely foreign to my ability, that we were not trying to smuggle into the city whiskey or tobacco. It was very amusing when we reached the frontier of Austria when the customs officer came through the car, to have them open up our baggage, overlooking things of importance, with the idea of finding a package of smoking tobacco, a few cigars or a small flask of whiskey. We were not allowed to carry over four ounces of whiskey, two ounces of tobacco and six cigars into Austria. We had a small bottle of whiskey, four ounces that had never been opened. The man was so persistent in searching our compartment that I remarked to our friends that I was sure he could smell it through the cork. Whether he could understand English or not I never knew, but he laughed heartily and left us. After having traveled for weeks through Italy and Switzerland, riding most of the time in carriages, we were not prepared to be driven through the streets of a great city of two million people on what seemed to us the wrong side of the street by a chauffeur who drove as a madman, in a car with the power and speed of a Fiat, the kind we had seen spin around saucerlike tracks at ninety miles an hour; but after turning corners on two wheels and skidding from first one side of the street to the other, we finally reached our hotel in safety. One could almost believe that he was running over some of the streets of Atlanta which were paved with Belgian blocks taken from Stone Mountain. Many of the most prominent streets in Vienna are paved with these rough stones.

One is impressed upon arriving with the attempt of these people to be kind and considerate; even though you understand very little they say and they understand less from you, they are constantly bowing and smiling to convince you, or try in their way to, that you are welcome. Imagine my dismay when getting a check cashed the clerk gave me a pocketful of silver and paper money for $20.00, and then in the course of a few minutes looked me up to say that he had given me only half enough. But after our first meal we found that to live in Vienna, we had practically enough for one day. I had often been told by many friends who had visited Europe that to live in Vienna was a great deal cheaper than in the States. While this is true, if you are willing to accept the accommodations they offer, to live as you are accustomed to, it is as expensive, or more so, than to live in New York. In addition to paying an exorbitant price for our dinner, we had to tip three men before leaving the dining room—the head waiter who does the bowing and takes your order, the waiter who brings the meal, and finally the man who clears the table; but with all of these the gratuities amount to about the same as you would give in a first-class restaurant or hotel at home.

The following morning I started out to locate the American Medical Association of Vienna. Was given very explicit instructions that with little trouble I could walk. But after a half hour's going in the direction as given, found myself back at the hotel. Realized then that the city is built in a circle. They have what is known as the outer and inner rings.

The administrative and aristocratic parts of the city are within the inner ring, while around the outer are the more modern portions with elegant buildings and beautiful streets. Finally I took a cab giving the driver the same instructions as I had. After going for some time, turning through many winding streets, we finally drew up in front of a very dilapidated building at 28 Schlossalgossa. Imagine yourself a perfectly innocent and unsuspecting American doctor in a foreign land looking for a street with such a name. In addition to paying my cabman his regular fare, was required to give a tip according to a register which amounted to about 15% of the tax. The American Medical Association is located on the second floor at the end of a winding stairway in three dark, dismal rooms. Even in a place like this one felt more or less at home and never did A. M. A. look so good. In a short while met many friends from the States and some from Atlanta who were all showing their loyalty by living in the American Pension. It was there we made arrangements to send our trunks.

It is a law of the continent of Europe, especially in Italy, Switzerland and Austria, that no buildings shall be over five stories high, and that it must be built of practically fire-proof material. Even in a city the size of Vienna we were told that they had not had a fire to which it was necessary to call a fire department for months.

In fact, I do not recall a fire department in Vienna. The streets for the most part in the old sections of the city are very narrow, often not having any side walks, this being true with the exception of the Circles and Boulevards, all of which are beautiful with shade trees and flowers all along. A very interesting little decoration consisted of flowers of the very gayest colors planted in boxes or frames and suspended up on the telegraph and telephone poles, these often fifteen and twenty feet up. The public buildings and grounds are beautiful; indeed, they far exceed anything in grandeur, beauty and number we have ever seen. Every few blocks are parks with flowers, seats and the ever present monuments and fountains. The people believe strongly in keeping alive the memory of queens, kings and heroes with everlasting monuments. They as a nation are lovers of fine arts, and especially of music, this having been the home of such men as Schiller, Wagner and Schubert. The King's Opera House is said to be one of the finest in the world. Through one of the parks they have built a water way which is said to have cost several million dollars. The banks of this in many places are grey granite covered with beautiful vines, mosses and flowers—in fact, a beautiful and romantic stream. The only thing lacking is the water, it having really been built to turn a part of the Danube river through, but this plan was afterwards found to be impracticable, so a small stream from the fountains of the city courses through this perfectly beautiful river bed.

The street car system is fine, in fact, as good as that of any of our large cities. One rule we soon learned to admire, that is, only as many persons as can find seats are allowed on a car, except that on each platform a certain number are allowed to stand, the number of seats and those allowed to stand being plainly marked on every platform. All cars have trailers.

We saw the wonderful houses of Parliament and the Emperor's homes. I say homes advisedly, for the palace in the city proper, including the gardens, covers several blocks and is said to contain 876 rooms; then there is his country home, Schonbrum, with 1,440 rooms, where he is for only a short stay each year. It was here that Napoleon made his headquarters part of the time that he was in Austria and that his nephew died in a room that is now occupied as the King's bed chamber. He has still another summer home with 1,100 rooms just outside of Vienna, where most of his time is spent. At each of these places are the most wonderful gardens and flowers, and too a regular army of workmen and soldiers on special guard. To be shown through one of these was to be shown such beauty and grandeur as we could not realize or believe a nation as poor as Austria could afford or support. We soon found that the rich in Austria became richer and the poor poorer.

It seemed strange to us who know nothing but freedom of thought and action to talk to these people and see how they were afraid to express an opinion on any public affairs. From the day of our arrival there was a constant discussion of the possibility of war with Servia. The House of Parliament was meeting with Emperor Joseph, who is also the Uncrowned King of Hungary. It was then generally believed that in the event war was declared, it would involve Germany, Russia and possibly France and England. The impression we got was that the people did not want to fight and that they were too poverty-tricken to support active warfare. The people as a whole are very poor. In addition to their poverty they are taxed so heavily that it is almost impossible for them to make or sell anything. Several doctors told me that it was much better to spend all one would make so as to keep the government from getting it. In the event one owns a piece of property that rents for $100.00 per month, the income tax on this amount would be $52.00, leaving the landlord $48.00 out of which he has to pay the regular taxes. They have inspectors to look over all one's private affairs, to say if you may or may not open a new boarding house or hotel, just how much you can afford to spend in attending the theaters, etc., and in the event your entertainments seem extravagant, you are promptly asked how you can afford to do so and so. The great number of inspectors, officers, etc., are all employed by the government and will some day draw pensions. Any one in the employ of the government in any capacity for ten years, draws a pension, this increasing with the number of years served. Wages paid to government employees are small, they, as in the case of street car conductors, are expected to live largely on tips. Even the washerwomen tip the conductors with one or two Hellers, which is equivalent to one-fifth of a cent. If you gave one of these men five Hellers, or one cent, he would stop the car and walk across the street to point out to you your destination.

The one most interesting thing in the city for us was the great University of Vienna with its Hospital of 5,500 beds. The customs of the people there will be more or less interesting to those who have not had the opportunity of visiting this country. From a moral standpoint the people are not very far removed from those of Italy. Vienna is supposed to be one of the most corrupt cities of the world. It was not unusual to see postcards of women in nude for sale on the streets. While we were in Vienna we were receiving the Atlanta papers which were carrying on a very animated discussion as to the advisability of men being allowed to wear bathing suits with the sleeves coming below the elbow. I was tempted to mail home post cards of the people in swimming showing their bathing suits, which consisted of a jockey-strop worn by the men and often no more than this worn by the women. It was not unusual to see a woman on the beach with nothing on but a sheet and she was not at all careful as to the use of this. There seemed to be nothing too vulgar for public display. There are a great many wine and tea houses. It seemed to me that when a building was opened to be occupied by one of these that the doors were removed or the keys thrown away. At all hours of the night the places were still open, and strange to say, doing a good business. It was not unusual to see great crowds on the streets at two and three o'clock in the morning. The army officers and soldiers are the most frequent visitors to these places. They have the most magnificent bands and the finest music. Even though Austria was supposed to be in mourning for the death of the Crown Prince, the gayety was such as we had never seen before. The soldiers impressed us as being very poorly provided for; they did not look well kept and groomed; their garrisons were wonderful, in many instances covering several blocks, but the odors from these, in especially two cases, are almost unbearable.

The old part of Vienna, and especially the poorer part, dates back from many years before the birth of Christ. There are a few things shown you, however, that date back further than the third and fourth centuries. The Roman Catholic church in Austria is much better preserved and in a more flourishing state than in Italy. Saint Stephen's church in Vienna is one of the most magnificent structures that had been our pleasure to see, but it looked sacriligious to see the poorest of the poor bringing and leaving at the feet of the Virgin their flowers and offerings of money. The buildings here are better furnished than those in Italy, being provided with seats and comforts of worship. There was a church located near our boarding place, the bell of which was often annoying. It rang a long or short time according to the financial ability of the person or the family of the person being buried. This church located just across the street from the hospital did a rushing business.

The first afternoon there we went for a walk and expected every moment to see the wonderful Krankenhause (Hospital). Not finding it, we appealed to an officer to direct us. He explained that the building along side of which we had walked for two blocks was the Medical Department of the University of Vienna and was the Hospital that we were looking for. After having traveled thousands of miles to see what I thought would be the most magnificent Medical University, or certainly one of the most magnificent, in the world, was not prepared to see a very old building, not over two stories high, that covered several acres. The buildings set aside for literary education and that of all science was wonderful to behold. They were surrounded in all instances by beautiful parks where the flowers are kept cultivated and bloom as hot house plants. The windows of the old hospital for the most part are very small and dusty. They reminded me of the joke of the Irish biddy who explained to her mistress that she only washed the windows on the inside so that the hated Jones children could not see in, but that they could see out. In fact, it seemed to us that if they ever had been washed, the date was so far back that there was no record kept. The newer part of the building, which has been completed within the last two years, is magnificent and the most modern hospital that it has ever been my pleasure to visit.

(To be Continued.)

